# Serum amylase on postoperative day one is a strong predictor of pancreatic fistula after pancreaticoduodenectomy: a retrospective cohort

**DOI:** 10.55730/1300-0144.5693

**Published:** 2023-08-11

**Authors:** Oğuzhan ÖZŞAY, Mehmet Can AYDIN, Salih Can ÇELİK, Kağan KARABULUT, Saim Savaş YÜRÜKER

**Affiliations:** 1 Department of Gastrointestinal Surgery, Faculty of Medicine, Ondokuz Mayıs University, Samsun, Turkiye; 2 Department of Gastrointestinal Surgery, Ordu State Hospital, Ordu, Turkiye; 3 Department of General Surgery, Faculty of Medicine, Ondokuz Mayıs University, Samsun, Turkiye

**Keywords:** Gland palpation, gland softness, pancreas texture, whipple

## Abstract

**Background/aim:**

Early identification of patients at risk for developing postoperative pancreatic fistula (POPF) after pancreaticoduodenectomy (PD) may facilitate drain management. In this context, it was aimed to examine the efficiency of the serum amylase (SA) value on postoperative day (PoD) 1 in predicting the occurrence of POPF.

**Materials and methods:**

A total of 132 patients who underwent PD were studied. Occurrences of POPF were classified according to the International Study Group on Pancreatic Fistula classification as a biochemical leak (BL) or clinically relevant grade b/c POPF (CR-POPF). Receiver operating characteristic analysis identified a threshold value of SA on PoD 1 associated with POPF formation.

**Results:**

Overall, 66 (50%) patients had POPF, including 51 (38.7%) with BL and 15 with CR-POPF (11.3%). The threshold value of SA associated with the development of POPF was 120 IU/L (odds ratio [OR]: 3.20; p = 0.002). In the multivariate analysis, independent POPF risk factors were SA ≥120 IU/L, soft pancreatic texture, and high-risk pathology (i.e., duodenal, biliary, ampullary, islet cell, and benign tumors); SA ≥120 IU/L outperformed soft pancreatic texture and high-risk pathology in predicting POPF, respectively (OR: 2.22; p = 0.004 vs. OR: 1.37; p = 0.012 vs. OR: 1.35; p = 0.018). In a subset analysis according to gland texture (soft vs. hard), patients with soft pancreatic texture exhibited a significantly higher incidence of POPF (63.4% vs. 34.4%) and SA ≥120 IU/L (52.1% vs. 27.9%); SA <120 IU/L had a negative predictive value of 82.5% for developing POPF in patients with hard pancreatic texture (OR: 4.28, p = 0.028).

**Conclusion:**

A SA value ≥120 IU/L on the day after PD, which is the strongest predictor for POPF, can be used as a biomarker of the occurrence of POPF. The advantage of SA measurement is that it can contribute to identifying suitable patients for early drain removal.

## 1. Introduction

Postoperative pancreatic fistula (POPF) is still a major concern after pancreaticoduodenectomy (PD) and can be categorized according to clinical severity [1]. Although low-grade biochemical leak (BL) is asymptomatic, it may progress to a clinically relevant grade b/c POPF (CR-POPF), which leads to subsequent morbidities such as hemorrhage, sepsis, abscess formation, and/or delayed gastric emptying [1–3]. Of note, early prediction of BL after PD also covers the occurrence of CR-POPF [1]; hence, a negative predictive factor(s) for BL can be helpful in an attempt to mitigate CR-POPF related morbidities, including postoperative intraperitoneal drain management.

An effort to mitigate these morbidities continues to ignite the debate on intraoperative intraperitoneal drain placement and postoperative removal timing. Prospective randomized evidence suggesting that intraoperative drain placement serves no benefit and even aggravates morbidity [4, 5] has been tempered by the early closure of a randomized study that assessed the same hypothesis [6]. For the majority of pancreatic surgeons, placing an intraoperative intraperitoneal drain is a routine component of PD, with concerns about maintaining control in the event of POPF development. Despite intraoperative drain placement being commonly done, prolonged remaining unnecessary drains might encourage POPF development due to negative pressure, erosion, and suction [7, 8]. Studies have indicated that early drain removal (on or before postoperative day (PoD) 3) in low-risk patients might reduce the incidence of POPF, subsequent abdominal complications, and healthcare costs compared to late drain removal (after PoD 3) [8–10].

Thus far, numerous POPF risk scoring systems have been presented based on well-known intraoperative-derived POPF risk factors, such as a small main pancreatic duct diameter, soft pancreatic texture, nonmalign pathology, and higher blood loss [2, 11, 12]. However, no consensus on the consequences of implementing these scores has yet been established in everyday clinical practice [13].

A straightforward measurement with a high predictive ability for POPF development in the early postoperative period may identify suitable patients for early intraperitoneal drain removal. As stated in the contemporary surgical literature, an elevated serum amylase (SA) value early after PD is a harbinger of POPF formation [10, 14–18]. However, limited knowledge exists about the predictive ability of SA for POPF in the current literature [10, 16]. Additionally, as a quantitative indicator of soft pancreatic texture, SA may be handy for POPF risk stratification in keeping with the postulate that gland texture stiffness cannot be identified by gland palpation during minimally invasive partial pancreatectomy.

Consequently, this study aimed to investigate the capacity of an SA on PoD 1 to predict the development of POPF in a cohort undergoing PD with intraoperative drain placement in an endeavor to identify patients suitable for early drain removal. The secondary goal was also to evaluate the association between the SA and endogenous POPF risk factors to facilitate POPF risk stratification in patients undergoing minimally invasive partial pancreatectomy.

## 2. Materials and methods

### 2.1. Study design and endpoints

This retrospective study included patients with malign or benign periampullary pathologies who underwent PD between January 2016 and July 2022. Individual patient data were collected from prospective databases maintained at the Department of Gastrointestinal Surgery of the Ondokuz Mayıs University Faculty of Medicine, Samsun, Türkiye. This study was approved by the Institutional Review Board of our hospital (approval number: 2022/414). All of the patients provided informed consent prior to their enrolment.

The primary endpoint of the study was the development of POPF, whereas the secondary endpoint was endogenous POPF risk factors.

### 2.2. Inclusion and exclusion criteria

The electronic charts of all of the patients who underwent PD, with or without concomitant venous resection, were reviewed. Patients with incomplete follow-up data and who experienced CR-POPF without previous BL were excluded from this analysis.

### 2.3. Data collection

Demographic, clinical, operative, and postoperative data were collected, including the pancreatic texture stiffness, main pancreatic duct (MPD) diameter, ≥1000 mL of blood loss, pancreaticojejunostmy (PJ) technique, need for venous reconstruction, SA values on PoD 1, the development of POPF, and final pathological report. Blood loss data were yielded from anesthesia charts and perioperative blood transfusion data. Pancreatic remnant texture stiffness (soft or hard) and MPD diameter (≤3 mm or >3 mm) were determined intraoperatively by the attending surgeon. Diagnoses other than pancreatic adenocarcinoma or chronic pancreatitis were considered high-risk pathology (i.e., duodenal, biliary, ampullary, islet cell, and benign tumors) [2]. All postoperative complications were classified according to the International Study Group for Pancreatic Fistula (ISGPF) [1], International Study Group for Pancreatic Surgery (ISGPS) [19, 20], and Clavien-Dindo classification [21]. Complications of ISGPF grades b/c and Clavien-Dindo grades 3–4 were considered CR-POPF and severe, respectively.

### 2.4. Perioperative management

All of the patients underwent the classical Whipple procedure. For most patients, the pancreatic remnant reconstruction method was PJ by the modified Blumgart technique, as previously described [22]. The polyethylene pancreatic stent between the MPD and jejunum and 2 nonvacuuming silicone intraperitoneal drains adjacent to the anastomoses were routinely placed. No patients were administered a prophylactic somatostatin analog before and during surgery. The nasogastric tube was set during the surgery and left in place.

According to institutional protocol, patients were followed-up in the intensive care unit during the early postoperative period. Most of the patients were taken to the clinic on PoD 1. Unless there were contraindications, the nasogastric tubes were removed on PoD 1 or 2. The drain output volume and content were tracked daily. Drain fluid was retrieved on PoD 3 from each patient and quantitatively analyzed for the SA content. When drain fluid SA on PoD 3 was more than 3 times the upper normal SA value of our institute (which is 100 IU/L), it was accepted as BL [1]. Finally, patients who were in the progression of BL to CR-POPF were also recorded.

### 2.5. Statistical analysis

Patients were dichotomized into 2 cohorts based on the existence of POPF, and all of the data were compared. Continuous variables presented as the median (interquartile range: IQR) or mean ± standard deviation were compared using t test or Mann-Whitney U test. Categorical variables were reported as numbers with percentages and compared using the chi-squared or Fisher exact test, as appropriate. The optimum PoD 1 SA threshold value associated with POPF formation was defined by receiver operating characteristic curve analysis (ROC) and expressed as the area under the curve (AUC). In addition, sensitivity, specificity, positive predictive values (PPVs), and negative predictive values (NPVs) were calculated, and the discrimination threshold was adjusted for easy clinical utilization. Univariate and multivariable logistic regression analysis (with backward variable selection) were performed to discover independent factors associated with the occurrence of POPF and an elevated SA value on PoD1 (dichotomized by the threshold value). In order to be used in the multivariable analysis, factors had to be statistically significant in the univariate analysis (p < 0.1). Results were presented as odds ratios (ORs) with 95% confidence intervals (CIs). P < 0.05 was considered statistically significant. All statistical analyses were performed using IBM SPSS Statistics for Mac 26.0 (IBM Corp., Armonk, New York, USA).

## 3. Results

### 3.1. Clinicopathological features

**Figure 1:**
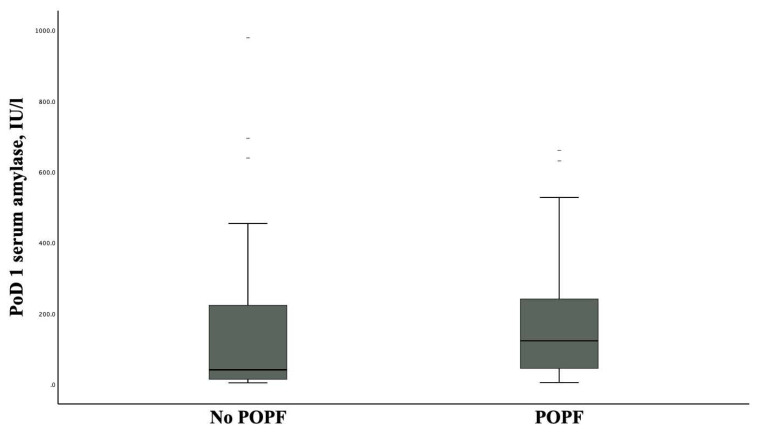
Distribution of the PoD 1 SA in patients with POPF and those without. The median PoD 1 SA was 41 IU/L (IQR: 14–224) in the patients with no-POPF and 123 IU/L (IQR: 43–202) in patients with POPF.

In the study period, a total of 151 patients underwent PD, and of these, 132 satisfied the inclusion criteria. Half of the cohort experienced POPF (51 [38.7%] BL vs. 15 CR-POPF [11.3%]). The demographic, operative, pathological, and postoperative data according to the presence of POPF are demonstrated in Table 1. Patients with POPF had a median SA value on PoD 1 of 123 IU/L (IQR: 43–242) compared to 41 IU/L (IQR: 14–224) in those without POPF (p = 0.001) (Figure 1).

### 3.2. Predicting pancreatic fistula using SA

**Figure 2:**
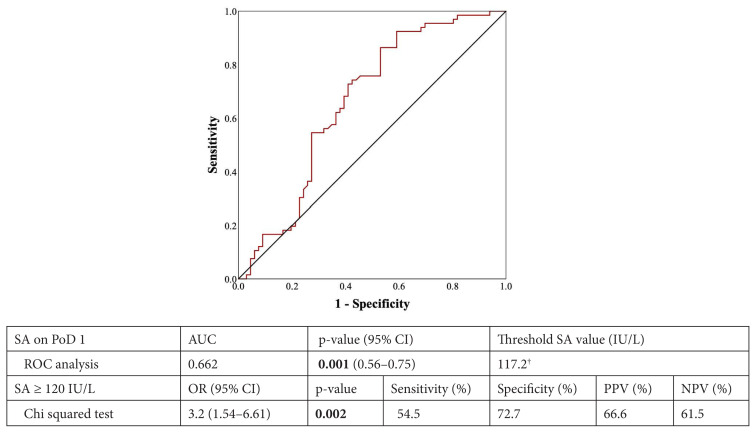
ROC analysis and chi-squared test revealed a significant association between the PoD1 SA and POPF. ROC: receiver operating characteristic, AUC: area under the curve, OR: odds ratio, CI: confidence interval PPV: positive predictive value; NPV: negative predictive value. † This value was corrected to 120 IU/L for easy clinical use and confirmed with the chi-squared test. Bold values indicate statistical significance.

A significant association was detected between the PoD 1 SA value and POPF formation in the ROC analysis (AUC = 0.662; 95% CI: 0.56–0.75; p = 0.001). The optimum SA threshold value of 117.2 IU/L associated with POPF formation was determined by the highest positive likelihood ratio (sensitivity/1-specificity). This value was corrected to 120 IU/L for clinical use and validated with the chi-squared test (OR: 3.20; 95% CI: 1.54–6.61; p = 0.002); sensitivity and specificity were 54.5% and 72.7%, respectively. Positive and negative predictive SA values ≥120 IU/L were 66.6% and 61.5%, respectively (Figure 2).

### 3.3. Risk factors of pancreatic fistula

Univariable and multivariate binary logistic regression analyses of the associations between clinicopathological characteristics and the development of POPF are summarized in Table 2. Soft pancreatic texture (OR: 1.37; 95% CI: 1.23–5.80; p = 0.012), high-risk pathology (OR: 1.35; 95% CI: 1.18–6.06; p = 0.018), and SA value ≥120 IU/L (OR: 2.22; 95% CI: 1.01–4.88; p = 0.004) were independent risk factors.

### 3.4. Predictors of postoperative elevated SA

The relationship between the POPF risk factors and ≥120 IU/L SA on PoD 1 is shown in Table 3. Only soft pancreatic texture (OR: 2.85; 95% CI: 1.35–6.02; p = 0.006) and high-risk pathology (OR: 2.74; 95% CI: 1.24–6.04; p = 0.012) were associated with ≥120 IU/L SA in the multivariate analysis.

### 3.5. Subset analysis based on pancreatic texture

In a subset analysis, patients with a hard pancreatic remnant texture had a lower rate of POPF (34.4%) compared with those with a soft texture (63.4%). An SA value ≥120 IU/L was determined in 27.9% patients with a hard pancreatic remnant texture, whereas it was found in 52.1% patients with a soft texture. An SA value <120 IU/L had an NPV of 82.5% for POPF formation in patients with a hard pancreatic remnant texture (OR: 4.28; 95% CI: 1.31–13.98; p = 0.028) (Table 4).

### 3.6. Postoperative complications and SA

Grades b/c delayed gastric emptying and the postpancreatectomy hemorrhage rate was 8.3% (n = 11). It was seen that 4 patients had CR-POPF-related hemorrhage, and 3 required a relaparotomy to control the hemorrhage. The rate of 90-day surgical mortality was 3.8% (n = 5). Moreover, 2 deaths occurred as subsequent postpancreatectomy hemorrhage, 2 following a postoperative myocardial infarction, and 1 after cranial embolism.

The association between a PoD 1 SA value ≥120 IU/L and subsequent complications is illustrated in Table 5. There was no association between a PoD 1 SA value ≥120 IU/L and CR-POPF and severe complications.

## 4. Discussion

The result of the current study indicated an association between soft pancreatic texture and high-risk pathology with an SA value ≥120 IU/l on PoD 1 after PD. Even though there have been efforts to create a quantitative marker for pancreas-inherent POPF risk factors (e.g., CT measurement, histomorphological evaluation on frozen section or resected PD specimen [23], direct measurement of pancreatic texture via Durometer [24]), the ISPGS has reported that the intraoperative assessment of pancreatic texture stiffness and MPD diameter by an experienced pancreatic surgeon has been adequate for POPF risk stratification [13]. Likewise, a metaanalysis emphasized intraoperative subjective judgment, which is acceptable for the assessment of pancreatic texture stiffness [25]. However, a quantitative marker that reflects the intraoperative-derived POPF risk factors may be more effective from a clinical standpoint in the minimal invasive pancreatectomy era, in which pancreatic texture stiffness is not determined by gland palpation. The present study suggests using an elevated SA value on PoD 1 as a quantitative indicator of soft pancreatic texture.

The most reasonable theory of postoperative hyperamylasemia after PD is the pooling of SA-rich pancreatic fluid at the PJ area due to PJ failure and its backflow into the blood vessels with increased tissue pressure [14, 15]. However, the SA increase timing is short; it is incompatible with extravasation from the PJ and reabsorption from the peritoneum [18]. This contradiction warrants further research. Another theory is that SA-rich fluid pooling in the pancreatic duct augments tissue pressure and pancreatic fluid backflow [14]. Therefore, a pancreatic stent placement between the MPD and jejunum during PJ may decrease pancreatic remnant tissue pressure, SA backflow, as well as the incidence of POPF.

The answer to why soft pancreatic texture is related to POPF formation contains many mechanisms: 1) higher exocrine function [26]; 2) smaller MPD diameter and a higher number of side branches [27]; 3) decreased suture-holding capacity, and increased ischemic or necrotic processes with suture compression [28]; and 4) the lower fibrosis degree and higher fat content of the pancreatic gland [29]. These factors can complicate PJ; hence, an elevated SA value just after PD reflects the unsuccessful PJ, namely the soft pancreatic texture. In summary, a high postoperative SA value can be considered robust evidence of a soft pancreatic texture.

Herein, independent POPF risk factors were an elevated SA value on PoD 1, soft pancreatic texture, and high-risk pathology. The SA threshold value on PoD 1 associated with POPF formation was 120 IU/L, as supported by the previous evidence [10, 14–17]. Additionally, an SA value ≥120 IU/L outperformed other independent POPF risk factors in predicting POPF formation (OR: 2.22; 95% CI: 1.01–4.88; p = 0.004). However, the present study failed to show that an MPD diameter ≤3 mm was associated with POPF, as noted by Okabayashi et al. [15]. This may be because of surgical technique improvements, institute volume, or utilized surgical loupe for duct-to-mucosal anastomosis [3, 30]. Furthermore, the recently introduced sequential POPF risk stratification system by the ISPGS presented a stronger association between soft pancreatic texture with POPF development than a small MPD diameter [13].

Numerous groups have presented data proving an elevated SA value as a marker for POPF development after PD [10, 14–18]. However, limited evidence has been served on the predictive capacity of the SA value for POPF development. According to Velu et al., a low PoD 0 SA value showed a low risk for POPF, even in patients with soft pancreatic texture [10]. In the current analysis, patients with a hard pancreatic remnant texture in addition to an SA value <120 IU/L carried the lowest risk for POPF (OR: 4.28; 95% CI: 1.31–13.98; p = 0.028; NPV: 82.5%). This high NPV may permit the choice of patients for postoperative early drain removal. As for its generalizability, this straightforward quantitative measurement yields evidence beyond the pancreatic texture in predicting POPF development.

The major limitation of this study was its retrospective and single-institution design. Additionally, an SA value ≥120 IU/L on PoD 1, it was not predictive for CR-POPF and severe complications despite the weighty evidence against it [10, 16–18]. Therefore, in the patient subset with an SA value ≥120 IU/L on PoD 1 will be required to analyze which additional parameters should be used to predict the progression of possible BL to CR-POPF. This conundrum is another essential limiting factor of the current study and the primary question of a planned subsequent investigation. Although an SA value ≥120 IU/L on PoD 1 was not specific to CR-POPF, it outperformed other risk factors in predicting POPF formation. The advantage of this predictive ability may be that it provides an additional contribution to the POPF risk score [2], which includes endogenous and intraoperative variables, as noted in recently published strong evidence [31] As it is known, the most important endogenous POPF risk factor is the soft pancreatic remnant texture. Ideally, intraoperative gland palpation by a senior surgeon is adequate to assess pancreatic remnant stiffness [13, 25]. In this context, the association of postoperative hyperamylasemia with a soft pancreatic remnant texture may facilitate POPF risk stratification following minimally invasive pancreatectomy. To the best of our knowledge, this is the first study to exhibit the association of a soft pancreatic remnant texture and postoperative hyperamylasemia in the literature. Moreover, the high NPV in the current analysis showed that patients with normal POD 1 SA values are at low risk of POPF formation [10, 14]. Therefore, those with a normal POD 1 SA value after PD may be candidates for the early removal of surgical drains. However, a prospective study pertaining to the consequences of early drain removal in patients with normal SA values after PD will be necessary to generate evidence to guide surgical drain management in patients undergoing PD.

In conclusion, the current study pointed out that an SA value ≥120 IU/L on PoD 1 after PD is a quantitative biomarker of soft pancreatic texture, high-risk pathology, and the development of POPF. This simple and routine measurement affords evidence beyond the pancreatic texture and high-risk pathology for predicting POPF. In addition, it may facilitate POPF risk stratification in the minimally invasive partial pancreatectomy era. Finally, an SA value <120 IU/L on PoD 1 after PD might provide justification for postoperative early intraperitoneal drain removal, especially in patients with a hard pancreatic texture.

## Figures and Tables

**Table 1:** Comparison of the clinicopathological characteristics between the patients with POPF† and those without.

POPF				
Characteristic	Overall (n = 132)	No POPF (n = 66)	POPF (n = 66)	p-value
Demographic				
Age (years), median (IQR)	64.5 (57–72)	64 (56–71)	65 (57–73)	0.418
Sex, female	61 (46.2%)	27 (40.9%)	34 (51.5%)	0.222
Preoperatively				
Previous surgery, yes	22 (16.7%)	9 (13.6%)	13 (19.7%)	0.484
BMI (kg/m^2^), ≥30	27 (20.5%)	12 (18.2%)	15 (22.7%)	0.666
Serum albumin (g/dL), mean ± SD	3.77 ± 0.52	3.87 ± 0.55	3.67 ± 0.48	**0.030**
Preoperative biliary drainage, yes	75 (56.8%)	32 (48.5%)	43 (65.2%)	0.053
ASA status, 3–4	13 (9.8%)	4 (6.1%)	9 (13.6%)	0.243
Comorbidities, yes				
Smoking	24 (18.2%)	13 (19.7%)	11 (16.7%)	0.821
Hypertension	58 (43.9%)	31 (47.0%)	27 (40.9%)	0.483
Coronary artery disease	22 (16.7%)	12 (18.2%)	10 (15.2%)	0.815
Chronic obstructive lung disease	13 (9.8%)	8 (12.1%)	5 (7.6%)	0.559
Diabetes mellitus	44 (33.3%)	24 (36.4%)	20 (30.3%)	0.580
Operative				
Pancreatic texture, soft	71 (53.8%)	26 (39.4%)	45 (68.2%)	**0.001**
Pancreatic duct diameter, ≤3 mm	50 (37.9%)	19 (28.8%)	31 (47.0%)	**0.031**
Venous reconstruction, yes	17 (12.9%)	10 (15.2%)	7 (10.6%)	0.603
Blumgart PJ, yes	96 (72.7%)	44 (66.7%)	52 (78.8%)	0.171
Intraoperative blood loss, ≥1000 mL	42 (31.8%)	24 (36.4%)	18 (27.3%)	0.350
Operation time (min), median (IQR)	420 (360–480)	408.5 (352.5–480)	420 (360–480)	0.321
High-risk pathology‡, yes	83 (62.9%)	34 (51.5%)	49 (74.2%)	**0.012**
Outcomes				
PoD 1 SA (IU/L), median (IQR)	75 (26–240)	41 (14–224)	123 (43–242)	**0.001**
Wound infection, yes	29 (22.0%)	14 (21.2%)	15 (22.7%)	1.000
Delayed gastric emptying§, yes	20 (15.2%)	6 (9.1%)	14 (21.2%)	0.089
Postpancreatectomy hemorrhage§, yes	18 (13.6%)	11 (16.7%)	7 (10.6%)	0.447
Severe morbidity¶, yes	17 (12.9%)	4 (6.1%)	13 (19.7%)	**<0.001**
90-day mortality, yes	5 (3.8%)	2 (3.0%)	3 (4.6%)	0.680

† Postoperative pancreatic fistula (POPF) was identified according to the International Study Group for Pancreatic Fistula criteria.

‡ High-risk pathology was indicated for all of the pathological diagnoses, except for pancreatic ductal adenocarcinoma and chronic pancreatitis.

§ Classified according to the International Study Group for Pancreatic Surgery criteria.

¶ Morbidities were graded using the Clavien-Dindo classification, and Grades 3–4 were considered severe morbidity.

IQR: interquartile range, PoD: postoperative day, BMI: body mass index, PJ: pancreaticojejunostmy, SA: serum amylase. Bold values indicate statistical significance.

**Table 2:** Association between the clinicopathological characteristics and postoperative POPF in patients undergoing PD (n = 132) using binary logistic regression analysis.

POPF							
		Univariate			Multivariate		
Characteristic	n	OR	95% CI	p-value	OR	95% CI	p-value
Preoperative serum albumin (g/dL)							
≥3.5	96	--	----	----	--	----	----
<3.5	36	1.57	1.15–5.73	**0.031**	1.01	0.83–4.86	0.121
Preoperative biliary drainage							
No	57	--	----	----	--	----	----
Yes	75	1.98	0.98–3.99	0.054	2.00	0.91–4.36	0.081
Pancreatic texture							
Hard	61	--	----	----	--	----	----
Soft	71	2.29	1.61–6.74	**0.001**	1.37	1.23–5.80	**0.012**
Pancreatic duct diameter							
>3 mm	82	--	----	----	----	----	----
≤3 mm	50	2.19	1.06–4.49	**0.033**	1.25	0.59–3.08	0.464
High-risk pathology							
No	49	--	----	----	----	----	----
Yes	83	1.71	1.30–5.64	**0.008**	1.35	1.18–6.06	**0.018**
PoD 1 SA (IU/L)							
<120	78	--	----	----	----	----	----
≥120	54	3.20	1.54–6.61	**0.002**	2.22	1.01–4.88	**0.004**

OR: odds ratio, CI: confidence interval. Bold values indicate statistical significance.

**Table 3:** Association between SA ≥120 IU/L on PoD 1 and clinicopathological characteristics of patients undergoing PD (n = 132) using binary logistic regression analysis.

PoD 1 SA ≥ 120 IU/L							
		Univariate			Multivariate		
Characteristic	n	OR	95% CI	p-value	OR	95% CI	p-value
BMI (kg/m^2^)							
<30	105	--	----	----	--	----	----
≥30	27	2.11	0.89–4.98	0.086	1.68	0.67–4.18	0.261
Pancreatic texture							
Hard	61	--	----	----	--	----	----
Soft	71	2.81	1.36–5.83	**0.005**	2.85	1.35–6.02	**0.006**
Pancreatic duct diameter							
>3 mm	82	--	----	----	--	----	----
≤3 mm	50	1.82	0.89–3.73	0.099	1.37	0.63–2.99	0.419
High-risk pathology							
No	49	--	----	----	--	----	----
Yes	83	2.70	1.25–5.81	**0.011**	2.74	1.24–6.04	**0.012**

OR: odds ratio, CI: confidence interval Bold values indicate statistical significance.

**Table 4:** Association between the pancreatic texture, SA, and POPF.

Soft pancreatic texture (n = 71)				
	PoD 1 SA < 120 IU/L	PoD 1 SA ≥ 120 IU/L		
n, (%)	34 (47.9)	37 (52.1)	n, (%)	p-value
POPF, no	15	11	26 (36.6)	0.312
POPF, yes	19	26	45 (63.4)	
Hard pancreatic texture (n = 61)				
	PoD 1 SA < 120 IU/L	PoD 1 SA ≥ 120 IU/L		
n, (%)	44 (72.1)	17 (27.9)	n, (%)	p-value
POPF, no	33	7	40 (65.6)	**0.028**
POPF, yes	11	10	21 (34.4)	

Bold values indicate statistical significance.

**Table 5:** Association between SA and postoperative complications.

	PoD 1 SA		
	<120 IU/L (n = 78 59.1%)	≥120 IU/L (n = 54, 40.9%)	
	n (%)	n (%)	p*-*value
POPF (ISGPF)†			
No	48 (61.5)	18 (33.3)	
Biochemical leak	23 (29.5)	28 (51.9)	**0.006**
Grade b/c POPF	7 (9)	8 (14.8)	
Postpancreatectomy hemorrhage (ISGPS)‡			
No	66 (84.6)	48 (88.9)	0.816
Grade a	5 (6.4)	2 (3.7)	
Grade b/c	7 (8.9)	4 (7.5)	
Delayed gastric emptying (ISGPS)‡			
No	67 (85.9)	45 (83.3)	0.754
Grade a	6 (7.7)	3 (5.6)	
Grade b/c	5 (6.4)	6 (11.2)	
Complications§			
No	21 (26.9)	12 (22.2)	0.518
Grades 1–2	49 (62.8)	33 (61.1)	
Grades 3–4	8 (10.3)	9 (16.7)	

† Classified according to the International Study Group for Pancreatic Fistula criteria.

‡ Classified according to the International Study Group for Pancreatic Surgery criteria.

§ Complications were graded with Clavien-Dindo classification.

Bold values indicate statistical significance.
